# Exploring Community Pharmacists’ Knowledge of Voluntary Assisted Dying and Authorised Disposal in Australia

**DOI:** 10.3390/pharmacy14040093

**Published:** 2026-06-26

**Authors:** Michelle Gyr, Heather Brown, Victoria Crisp, Milan du Plooy, Noora Al Hasooni, Natalia Popowicz, Liza Seubert, Tanya Burgess

**Affiliations:** 1 Department of Pharmacy, School of Health and Clinical Sciences, University of Western Australia, Crawley, WA 6009, Australia; heather.brown@health.wa.gov.au (H.B.); victoria.crisp@health.wa.gov.au (V.C.); milan.duplooy@health.wa.gov.au (M.d.P.); 23765676@student.uwa.edu.au (N.A.H.); liza.seubert@uwa.edu.au (L.S.); 2 WA Voluntary Assisted Dying Statewide Pharmacy Service, North Metropolitan Health Service, Nedlands, WA 6009, Australia

**Keywords:** Voluntary Assisted Dying (VAD), community pharmacists, knowledge, disposal, assisted dying

## Abstract

Background: Voluntary Assisted Dying (VAD) legislation in Western Australia (WA) introduced new responsibilities for community pharmacists, including the safe disposal of unused VAD substances. Pharmacists may receive VAD-related enquiries; however, their preparedness, including their knowledge of VAD, remains largely unexplored. Aim: To explore Western Australian community pharmacists’ knowledge of VAD and their role as Authorised Disposers under the Voluntary Assisted Dying Act 2019 (WA). Methods: Community pharmacists in WA were invited to participate in an anonymous, online survey consisting of multiple choice and open-ended questions. Results: Of 143 respondents, 76% were aware that VAD is an end-of-life option; despite this, 64% were uncertain about its relevance to their role. Almost one-third had received VAD-related enquiries. Of these, 40% could not provide information, with half attributing this to insufficient knowledge. Among those yet to receive a VAD-related enquiry (*n* = 115), 75% felt ‘not confident’ to respond, with 76% attributing this to lack of knowledge. A total of 63% of participants were unaware that they may be requested to perform authorised disposal. Uncertainty surrounding the process and the legal and ethical aspects were frequently mentioned. Conclusion: WA Community pharmacists demonstrate awareness of VAD legislation but have gaps in knowledge, particularly regarding authorised disposal. Further training and resources are essential.

## 1. Introduction

Voluntary Assisted Dying (VAD) is the legal process that allows eligible individuals with a terminal illness to voluntarily request and receive prescribed medication to end their life [1]. In 2021, VAD came into effect in Western Australia (WA) under the VAD Act 2019 (WA). The Act designates Australian Health Practitioner Regulatory Agency (AHPRA) registered pharmacists as Authorised Disposers, responsible for the safe disposal of unused or remaining VAD substances [2]. In WA community pharmacies, the Authorised Disposer is deemed to be the pharmacist who holds overall responsibility of the pharmacy or the Pharmacist-in-Charge (or Pharmacist Manager). Authorised disposal occurs when an unused or remaining self-administered VAD substance is given to an Authorised Disposer [2]. Community pharmacists may be amongst the most accessible health professionals who patients or caregivers approach with VAD enquiries and about authorised disposal [3]. Despite this, the extent of pharmacists’ preparedness for these responsibilities, particularly within community pharmacy settings, remains largely unexplored.

A consistent theme within the literature is that pharmacists report inadequate VAD knowledge and training. Several international studies have identified significant gaps in pharmacists’ understanding of legislation, procedural requirements and general knowledge of VAD [4,5,6,7]. A survey of New Zealand pharmacists found that 83% had not received any training relating to VAD, and only half rated their knowledge as “somewhat knowledgeable” [7]. Similar gaps were reported in Canadian studies, where a mixed-methods study involving over 600 pharmacists found that 75% had received little to no Medical Aid in Dying (MAID)-related education [8]. Additional Canadian studies similarly identified that many pharmacists feel inadequately prepared to handle MAID-related situations due to limited training and ambiguous professional guidelines [5,6]. In Australia, where research on pharmacists’ roles in VAD remains scarce, Isaac et al. (2019) [4] found that pharmacists expressed uncertainty about their role and highlighted the need for further education on the topic. The study found that the majority of the 40 participants expressed the need for the development of training courses and learning opportunities to enhance their understanding of VAD [4]. Through a structured literature review, only five studies globally were identified that specifically assess pharmacists’ knowledge of VAD, as all other studies researched heavily into attitudes and perspectives regarding VAD that did not align with this research aim. Despite the limited number of studies, all revealed clear interest in receiving additional education to increase their knowledge of VAD as a key theme [4,5,6,7,8].

There remains a notable lack of research specific to the Australian context. Although the legal framework and scope of practice differ between countries and jurisdictions, pharmacists globally face similar professional, ethical and practice issues when new legislation, such as introducing VAD, expands their responsibilities. Geography and population distribution in WA can make equitable healthcare particularly challenging due to its large size and remote communities. Moreover, few studies have specifically examined community pharmacists, despite their potential role as the first points of contact for VAD-related enquiries and authorised disposal. Of the limited studies available, most were conducted overseas (Canada and New Zealand), and often included mixed participant groups, such as technicians or pharmacy students, reducing the relevance of findings to practicing pharmacists [6,7]. Furthermore, existing research often focused on emotional burden or personal beliefs rather than knowledge [4].

Little is known about how pharmacists acquire knowledge related to VAD, how they respond to VAD enquiries, or what professional barriers they may face. Additionally, due to the lack of education and training revealed in studies, pharmacists could be unaware that they may inherently hold the role of Authorised Disposer.

As VAD becomes increasingly integrated into end-of-life care in WA, identifying the knowledge gaps, preparedness and support needs of community pharmacists is critical to ensuring safe delivery of care and disposal of VAD substances. This research is essential not only to inform future education and policy frameworks, but also to support community pharmacists in confidently fulfilling their role, including as an Authorised Disposer, thereby improving the quality of information and support available to patients and their families during a highly sensitive time.

This study aims to explore Western Australian community pharmacists’ knowledge of VAD and their role as Authorised Disposers under the VAD Act 2019 (WA).

## 2. Methods

The survey (Qualtrics XM, Version 2025, Provo, UT, USA) was distributed to registered community pharmacies in WA via email in August 2025. Researchers developed each survey question, mapping to the study’s aims and objectives. The survey contained both multiple-choice questions and free-text responses across four domains: (1) general information, (2) VAD enquiries, (3) authorised disposal, and (4) VAD training and education. Given the sensitivity of the topic—as it involves decisions about the end of life, which is deeply personal and often tied to strong ethical, cultural, religious and emotional beliefs—multiple-choice questions were either forced-response or requested-response. Thus, not all questions were completed by the total number of participants who attempted the survey, and these incomplete surveys were not excluded. Likewise, the free-text responses were optional.

To improve face validity, survey questions were revised following feedback from seven University of Western Australia (UWA) pharmacy academics, a cohort of UWA pharmacy students and two pharmacists from the WA VAD Statewide Pharmacy Service. The survey was piloted with a group of 15 pharmacy students and 5 pharmacy academics to assess comprehension, skip logic and survey flow, with revisions made based on comments.

Eligible participants were Australian Health Practitioner Regulation Agency (AHPRA) registered pharmacists and provisionally registered intern pharmacists who had worked in community pharmacy in WA within the previous four years, aligning with the commencement of VAD in WA in July 2021 [1]. A list of 698 registered pharmacies in WA was obtained from the Pharmacy Registration Board of Western Australia’s website [9]. An internet search was conducted to procure the email addresses of pharmacies, and those whose emails were not available were contacted via phone call. In addition to emailing the survey to pharmacies, participants were recruited through researchers’ professional networks. To extend the sample, snowball sampling was utilised, with participants invited to share the study information with eligible colleagues. To capture participants from outside metropolitan areas, 156 posters were mailed to rural community pharmacies across WA, with ‘rural’ defined as located outside the Perth metropolitan area. In addition, UWA Pharmacy cohort students distributed posters to community pharmacies within metropolitan areas. Recruitment was also supported via advertisements on social media, using the researchers’ LinkedIn accounts and relevant Facebook groups. A target sample size of 93 participants was determined via the Australian Bureau of Statistics sample size calculator, using a confidence level of 95%, margin of error of ±10%, and a total population of 2643. As the number of community pharmacists in WA is not publicly reported, an estimate was derived from the total number of registered pharmacists. In 2024, 4237 pharmacists were registered in WA [10]. A proportion of 62.4% was applied to approximate those in community practice, consistent with a previously published workforce analysis on principal place of practice [11].

The UWA Ethics Committee approved this study (ET000339). Consent was obtained via a Participant Information and Consent Form at the start of the survey. Participants who did not provide consent were excluded.

Descriptive statistics, generated within Qualtrics, were used to summarise survey responses. All categorical data was expressed as frequencies and percentages. R: A Language and Environment for Statistical Computing software, Version 4.5.3, was used to perform some additional analysis. Pearson’s chi-squared test (with Yates’ continuity correction for comparisons that were 2 × 2) was used to compare categorical responses between regional and metropolitan participants. A *p*-value of <0.05 was considered statistically significant. Following data immersion, four researchers independently coded the data, and two researchers reconciled differences through discussion until a consensus was met. An inductive approach to thematic analysis with content analysis as described by Kleinheksel was used to analyse open-ended questions [12].

## 3. Results

Of 698 registered pharmacies in WA, 695 were successfully contacted via email. However, as participants were also recruited through researcher professional networks, snowball sampling and social media channels, the total number of individual pharmacists reached could not be determined and a traditional response rate could not be calculated. A total of 163 community pharmacists participated in the survey.

### 3.1. Demographics

Most respondents were female (66%), and the largest proportion were aged between 40 and 49 years (31%), followed by 20–29 years (29%); see Table 1. The majority practiced in metropolitan areas (69%). Of the rural respondents (31%), the Southwest was the most represented rural area (12%). In terms of primary roles, most participants identified as pharmacists (36%), pharmacists in charge (24%), or pharmacy owners (29%). Nearly one-third of respondents had been practicing for more than 20 years (29%), and most worked more than 30 h per week (71%), reflecting a sample with extensive experience and active involvement in community pharmacy practice.

### 3.2. VAD Awareness

**Figure 1:**
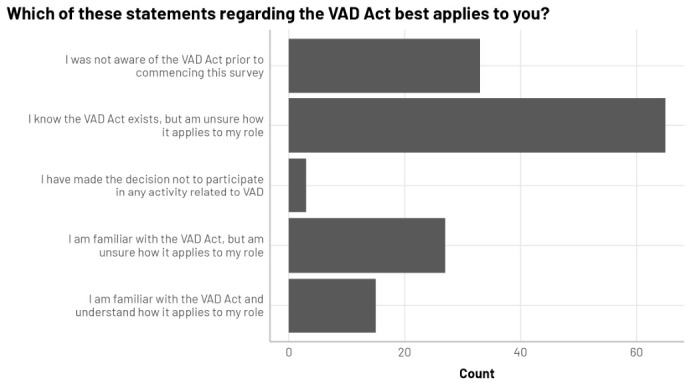
Community pharmacists’ knowledge of the VAD Act.

Participants were asked whether they were aware that VAD was an end-of-life option prior to commencing the survey. Of the 163 pharmacists who participated in the survey, a total of 143 opted to provide a response to this question. A total of 76% (*n* = 109) were aware that VAD is an end-of-life option available for eligible Western Australians. When asked about the initial source of their knowledge, 99 participants provided a free text response. Of these, 36 responses cited the media (including news and social media), 11 reported first learning about VAD from a patient and nine mentioned work colleagues. The majority of respondents were familiar with or aware of the VAD Act 2019 (WA) but were uncertain how it applies to their role as community pharmacists (64%, *n* = 92), while 23% (*n* = 33) were not aware of the Act prior to commencing the survey (Figure 1.). Of the metropolitan respondents, 78% indicated they were aware that VAD is an end-of-life option in WA, while 73% of rural participants indicated they were aware. Statistical analysis suggested no significant difference between rural and metropolitan participants’ awareness of VAD being an end-of-life option (*p* = 0.73).

### 3.3. VAD Enquiries

**Figure 2:**
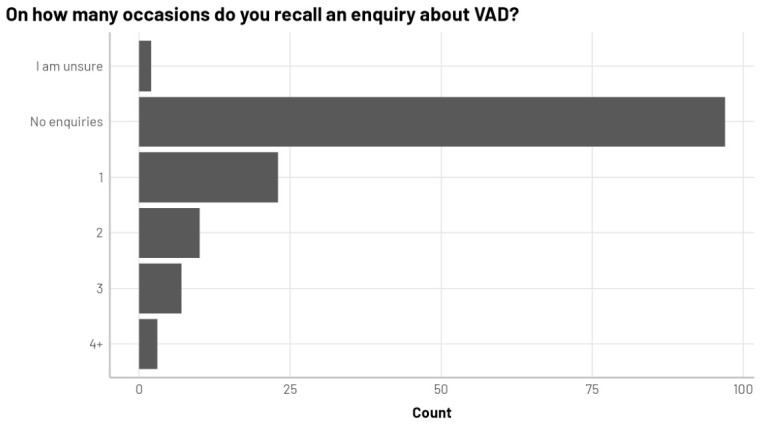
VAD enquiries received by community pharmacists.

Of the 163 returned surveys, 142 participants opted to respond to a question related to VAD enquiries. A total of 30% (*n* = 43) reported having received at least one VAD-related enquiry within a community pharmacy, while two reported they had but were unsure of how many (See Figure 2). Of these, 29% (*n* = 13) of rural participants indicated they had received one or more enquiries compared with 33% (*n* = 32) of metropolitan participants, a difference that was not statistically significant *p* = 0.09.

**Figure 3:**
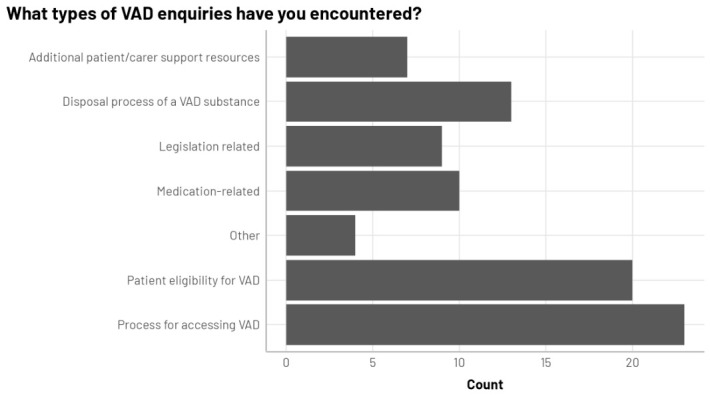
Nature of VAD enquiries.

When asked who initiated the discussion, 24 responses reported that the enquiry was raised by a person seeking information or intending to access VAD, while 28 indicated a carer of a person seeking information or intending to access VAD. Other responses included enquiries from colleagues and other healthcare professionals. The nature of the VAD enquiries most commonly related to the process for accessing VAD (51%, *n* = 23) and patient eligibility (44%, *n* = 20), followed by disposal process of VAD substance (29%, *n* = 13). Other enquiries related to medication efficacy, side effects and administration, legislation and additional patient/carer support resources. See Figure 3.

Among those who had received an enquiry (*n* = 45), 40% (*n* = 18) reported being unable to provide information to the enquirer, with half of these (*n* = 9) stating this was related to a lack of knowledge of VAD. Of the rural participants who had received one or more enquiries, 69% (*n* = 9) were unable to provide information while 28% (*n* = 9) of metropolitan participants reported being unable to provide information. Further statistical analysis suggested significance (*p* = 0.03).

When asked how confident would you feel providing VAD information if the topic were raised by a patient or carer, out of 115 responses, 75% (*n* = 86) reported that they would feel ‘not confident’ in responding.

Overall, 66 responses to the open-ended questions mentioned a perceived lack of knowledge regarding VAD. Most commonly participants described a lack of knowledge relating to the process of VAD, such as eligibility criteria and access pathways (16 responses). One participant noted that they had “limited knowledge about the process.” Participants frequently reported uncertainty about the legal and ethical requirements in their role (10 responses), one suggesting that they were “not comfortable with answering the questions as not sure about the legalities around it”. Similarly, uncertainty around the scope of practice of community pharmacists in VAD (10 responses), illustrated by the comment “I support VAD but am unsure of my role”. Seven responses indicated an awareness of referral pathways for patients seeking VAD.

### 3.4. Authorised Disposal

The majority of participants were unaware that registered pharmacists in WA are assigned the role of Authorised Disposer under the VAD Act 2019 (WA) and that they may be requested to fulfil this role (63%, *n* = 82). Pharmacists most commonly reported uncertainty around the process of disposal and the legal and ethical implications involved in acting as an Authorised Disposer (33 and 13 responses, respectively) in open-ended questions. When asked about factors impacting the ability to act as an Authorised Disposer, one participant responded, “insufficient resources available for disposal” and another “not knowing next steps or not being able to achieve them in practice.” Furthermore, limited knowledge regarding the medication used in VAD, particularly relating to the safe handling of the substance, was frequently reported (15 responses). One response suggested a greater willingness to perform the role of Authorised Disposer provided they receive more information on safe handling, such as “adequate warnings on toxicity etc.” Several responses indicated awareness that disposal of a VAD substance would fall within the scope of practice of pharmacists (5 responses). Insufficient training and education were the most commonly mentioned reason, attributed to a lack of knowledge on authorised disposal (30 responses), followed by a lack of guidelines and resources (14 responses). Eight participants reported that they had acted as an Authorised Disposer. Five of these stated they did not feel confident fulfilling the role; however, all eight indicated they would do it again if asked. Authorised Disposers were asked which resource(s) were accessed to assist them. Six respondents indicated they had contacted the VAD Statewide Pharmacy Service, while five referred to the instruction sheet that is contained within the VAD kit. Other sources of information included the Department of Health website (*n* = 1) and work colleagues (*n* = 1).

### 3.5. VAD Training and Education

A total of 138 of the 163 participants opted to answer questions relating to training and education, with 92% (*n* = 127) indicating they had not received any training or education relating to VAD in WA. In open ended questions, community pharmacists commonly attributed their lack of knowledge around VAD to insufficient training and education (11 responses), along with lack of guidelines and resources (11 responses). Of the 8% (*n* = 11) that indicated they had received some form of VAD training, 91% (*n* = 10) reported that training and education had improved their confidence to respond to VAD enquiries.

## 4. Discussion

Pharmacists’ roles are expanding across the world, and this study highlights the importance of ensuring pharmacists are provided with training for new areas of practice and changes in legislation. This study aimed to explore community pharmacists’ knowledge of VAD and their role as Authorised Disposers in WA.

The results suggest that while the majority of pharmacists (76%, *n* = 109) were aware that VAD is legal in WA, there is uncertainty about how the legislation applies to practice. Awareness of VAD legislation was similar for both rural- and metropolitan-based pharmacists (73% and 78%, respectively). The finding that 30% of participants had received a VAD-related enquiry shows that patients and caregivers are approaching community pharmacists for information in this area. With 29% of rural participants reporting that they had received one or more enquiries compared with 33% of metropolitan participants, the findings suggest a comparable reliance on community pharmacists for VAD information across both rural and metropolitan areas.

This reflects the expectation set out in the Advanced Pharmacy Australia (AdPha) 2025 Pharmacy Practice Update, which advises that pharmacists should anticipate conversations about VAD arising in discussions with people who are at or approaching the end of their life [13]. This highlights the importance of pharmacists having sound knowledge to provide accurate and supportive care.

Pharmacists, like all healthcare professionals in WA, do not have to participate in VAD, but they should respect the choices patients make about their end-of-life care, even if it does not align with their own values. Under the VAD Act 2019 (WA), pharmacists can provide information if asked but they are unable to initiate VAD discussions with patients [14]. Whilst community pharmacists do not have to have detailed knowledge about eligibility, assessment, prescribing or administration processes, they are delegated the role of Authorised Disposer in the legislation [2,14]. All community pharmacists should know how to respond appropriately and how to refer patients seeking information or access to VAD. Community pharmacists with overall responsibility should be aware that they may be asked to perform authorised disposal and what is involved if they accept this role or be prepared to respectfully refer to another pharmacist or pharmacy in the case of conscientious objection.

### 4.1. Knowledge of VAD

Many participants perceived themselves as lacking knowledge about VAD, with most reporting that they had not yet received a VAD enquiry (*n* = 97) and 75% of participants expressing low confidence in responding to one. Of the 45 participants who indicated they had received at least one VAD related enquiry, 40% were unable to provide information to the enquirer. Less rural pharmacists were able to provide information to enquirers than metropolitan pharmacists, at 69% and 28%, respectively. This raises some concerns over patients being able to access timely and accurate information when making complex end-of-life decisions. Delays or misinformation could adversely affect patient safety, wellbeing and continuity of care. Rural patients may be more disadvantaged; however, due to the low numbers and without deeper exploration a conclusion cannot be made.

When exploring specific areas of knowledge, the most common gaps were related to the process of VAD, as well as the legal and ethical responsibilities tied to their role and the overall scope of practice within the VAD framework. This shows that simply knowing VAD is legal does not necessarily mean pharmacists have the practical knowledge or skills needed to effectively support patients, caregivers or other healthcare professionals in practice.

The main contributors to a lack of knowledge were reported to be the limited number of guidelines and accessible resources and insufficient training and education. This aligns with the current context in WA, where pharmacists have access to a limited set of community pharmacy-specific VAD resources. These include the VAD Substance Disposal Guidance for Pharmacists provided by the WA VAD Statewide Pharmacy Service [15] and the 2025 Pharmacy Practice Update on Voluntary Assisted Dying, published by Advanced Pharmacy Australia (AdPha) [13].

Despite VAD being implemented in Western Australia in July 2021, the findings of this study suggest that education and training for community pharmacists have not been consistently embedded into practice over the subsequent four years. This may reflect several system-level challenges. There are approximately 700 registered community pharmacies distributed across a geographically vast state, creating logistical challenges for the delivery of uniform training and support. In addition, engagement from professional pharmacy bodies and advocacy groups in relation to community pharmacy-specific VAD education has been limited.

While some initiatives have commenced, including the development of online learning packages and targeted communication with pharmacies nominated for authorised disposal, these efforts are still emerging and may not yet have achieved widespread reach or uptake. Furthermore, the relatively low frequency but high-stakes nature of VAD encounters may contribute to lower prioritisation of training in busy community pharmacy environments. Together, these factors may explain the knowledge gaps observed in this study despite the legislative framework being in place for several years.

Accessible resources and professional guidelines may enhance pharmacists’ knowledge of VAD and promote clarity on their role, enabling respectful, supportive conversations with patients and caregivers on a sensitive topic like VAD [13]. A cross-sectional nationwide study revealed that the majority of participants (including pharmacists, intern pharmacists and pharmacy students) perceived professional guidance resources as ‘very useful’, reporting that these resources helped them familiarise themselves with content and update their knowledge in a particular domain of practice [16]. This suggests that pharmacists could potentially benefit from a pharmacy-specific VAD resource [13]. Additionally, previous research suggests that lack of training may leave health professionals feeling unprepared to respond to VAD requests, creating a potential barrier to engaging in VAD-related conversations with patients and possibly affecting the therapeutic relationship [17]. Moreover, survey results show a small number of pharmacists reported receiving formal training and education which positively influenced their knowledge around VAD.

These knowledge gaps should be addressed as pharmacists are often the first point of contact for patients and caregivers seeking guidance during and outside of regular hours, making it important that they provide accurate information [3]. Nationally, pharmacists are among the most accessible healthcare professionals, with over 2000 pharmacies open after hours, including on weekends, and more than 443.6 million patient visits to community pharmacies each year in Australia [18]. Furthermore, in the context of WA’s geographical scale, rural pharmacists may serve as a particularly accessible point of contact for those seeking guidance on VAD. From July 2021 to June 2025, 3779 patients made a first request for VAD in WA. Of these, 25.8% were located in a regional area [19]. Thus, it is important that pharmacists across WA are equipped with the knowledge to respond to VAD enquiries, as their accessibility positions them to provide timely support and guidance.

### 4.2. Knowledge of Authorised Disposal

Pharmacists demonstrated a lack of knowledge of their role as Authorised Disposers, with 63% of respondents indicating they did not know they could be asked to act in this capacity. When participants were asked about specific aspects of authorised disposal, the most frequently reported gaps were related to unfamiliarity with the disposal process, uncertainty around the legal and ethical responsibilities and limited knowledge of how to safely handle VAD substances. These gaps raise concerns about pharmacists’ ability to perform the role of Authorised Disposer safely and with confidence.

Since the introduction of VAD in WA, demand for VAD kits has risen significantly, with a 60.8% increase in total supplies over the last reporting period (July 2024 to June 2025) [19]. This growing demand increases the likelihood that pharmacists will be approached with enquiries or requests for authorised disposal.

As of 30 June 2025, 1368 kits have been distributed in WA since the service began in July 2021, including both practitioner- and self-administered VAD kits [19]. In WA, an eligible person makes an administration decision whereby a suitable administration protocol is chosen. For self-administration protocols, pharmacists are responsible for disposing of any unused VAD substances, which may occur if a decision is revoked or a person dies from other causes [14]. For practitioner-administered protocols, the administering practitioner is responsible for disposing of any unused VAD substances [14].

Of the 163 pharmacists who responded to the survey, 8 indicated they had acted as an Authorised Disposer. There were 668 administration decisions made in WA between July 2024 and June 2025. Of these, 9% elected for self-administration and 91% chose a practitioner-administered protocol [19]. Given the smaller proportion of self-administration decisions, the survey captured a meaningful proportion of pharmacists with experience as Authorised Disposers. This strengthens the study’s credibility to assess WA community pharmacists’ knowledge of authorised disposal.

The majority of participants were unaware that they may be asked to act as an Authorised Disposer. Correct disposal is essential to protect public safety and prevent potential misuse of medicines, including the VAD substance, meaning that any uncertainty poses risk [20]. Interestingly, a common theme that emerged from the data was a lack of knowledge on the safe handling of VAD medicines. Pharmacists stated that they are less willing to dispose of a VAD substance if they think it is unsafe to handle. Consequently, this may prevent pharmacists from accepting VAD kits for disposal. It is suspected that this could cause inconvenience and distress to grieving family members or caregivers who have the responsibility of bringing any unused VAD substances to an Authorised Disposer.

These findings highlight important operational implications for community pharmacy practice. Uncertainty regarding authorised disposal extends beyond knowledge gaps and reflects practical challenges related to workflow integration, storage and occupational safety. The storage, handling and disposal of VAD medications require clear guidelines to support pharmacists in ensuring safe and legally compliant practice.

Further studies should investigate the barriers and enablers that may impact pharmacists’ ability to respond to VAD enquiries in the community pharmacy setting in both rural and metropolitan locations, as well as factors that may improve participation in authorised disposal. This may be achieved through qualitative research, such as interviews and focus groups. Taken together, these findings should direct the development of targeted training and clear guidelines to support pharmacists in both responding to VAD enquiries and fulfilling their role as Authorised Disposers. Understanding the barriers and enablers will allow professional pharmacy bodies to allocate resources and tailor training and education effectively to the needs of all community pharmacists. Any VAD community pharmacist training or educational interventions would benefit from follow-up research to assess the impact on VAD knowledge and confidence to respond to VAD related queries.

## 5. Strengths

A strength of this study is the diversity of participants, spanning across a range of ages, gender, professional roles, levels of experience and geographic locations. A total of 66% of participants were female and, as per AHPRA’s 2024 annual report, the proportion of registered pharmacists in WA that were female was also 66% [10]. The distribution of participant age also closely reflects published data on Australia’s pharmacists [10]. In the sample population, 69% of pharmacists practiced in metropolitan areas and 31% in rural settings. The distribution closely reflects the broader Western Australian pharmacy landscape, where in 2017, 71% of registered pharmacies were located in metropolitan Perth and 29% were rural [21]. This demographic alignment supports the representativeness of our sample to the WA pharmacist population. Finally, this study contributes valuable insights to an area where there is currently limited research, addressing a significant gap in the literature on pharmacists’ knowledge with VAD [4,5,6,7,8].

## 6. Limitations

Eligibility screening was implemented at the beginning of the survey to prevent additional pharmacy staff (e.g., hospital pharmacists, pharmacy technicians, assistants or students) from responding to the survey. Despite this, the possibility that some respondents held roles other than that of community pharmacist cannot be eliminated and is a limitation of this study. Attempts were made to mitigate this by including an initial screening question to explicitly confirm participants’ current role as a community pharmacist, thereby reducing the likelihood of ineligible participants completing the survey. Due to the exploratory and descriptive nature of our study, we acknowledge that psychometric validation of the survey was not undertaken. The instrument was designed for the purpose of capturing knowledge and practice matters rather than as a validated psychometric scale.

Considering the nature of the topic and its recent legalisation, the potential for sample bias is likely. The proportion of pharmacists who responded is low compared to the number of pharmacies contacted. Both self-selection bias and non-response bias are limitations and may skew the study sample towards pharmacists who have had experience with VAD or who have strong opinions on the topic, while those with little to no awareness may have been less motivated to respond. Therefore, it is possible that the knowledge that community pharmacists have regarding VAD and authorised disposal reported in the study may be higher than that in the general population and the true knowledge gap may be underestimated. This strengthens the study’s conclusions, that even among a potentially engaged pharmacist population, substantial gaps in VAD knowledge and preparedness were identified, reinforcing the need for targeted training and resources.

Direct contact with individual community pharmacists was not possible, as only the email addresses of registered pharmacies in WA are publicly available. Consequently, surveys were distributed to pharmacies, with the expectation that any pharmacists present would have the opportunity to participate. Despite efforts to verify addresses, several emails were returned as undeliverable.

Due to the anonymity of the survey, it was not possible to identify whether multiple pharmacists from the same pharmacy responded to the survey; therefore, it is possible this could have resulted in some bias if multiple surveys were returned from the same pharmacy or pharmacy chain. Despite this, each individual pharmacist brings their own knowledge, perspectives and experience.

The results are limited to registered community pharmacists in WA and may not be generalisable to other states or jurisdictions due to differences in legislation; this highlights the challenges of translating new legislation into confident and competent practice, which may similarly be encountered wherever similar services are introduced or expanded.

## 7. Conclusions

This study is the first to explore the knowledge gap among community pharmacists regarding VAD and their role as Authorised Disposers in Australia. WA community pharmacists reported lacking knowledge in the domain of VAD while expressing a desire for VAD education. There is a need for clearer support from health authorities and professional bodies (i.e., in the form of training and education) to address this gap.

Although VAD enquiries and disposals are still relatively uncommon in WA, the rapid increase in demand for VAD means pharmacists must be prepared to respond to VAD enquiries and to act as Authorised Disposers through appropriate training and education.

This study also contributes new knowledge to the literature by identifying training and education as the most important enablers of knowledge among community pharmacists. Therefore, it identifies the need for jurisdiction-specific educational programs for pharmacists, as well as the development of clear guidelines and resources to standardise practice.

## Figures and Tables

**Table 1:** Demographics of survey participants.

Characteristics	Number of Participants (%)
Gender (*n* = 157)	
Female	103 (66%)
Male	53 (34%)
Prefer not to answer	1 (1%)
Age Range (years) (*n* = 154)	
20–29	44 (29%)
30–39	41(27%)
40–49	49 (31%)
50–59	14 (9%)
60+	6 (4%)
Place of Practice (*n* = 150)	
Metropolitan	103 (69%)
Rural	47 (31%)
Goldfields	3 (2%)
Great Southern	7 (5%)
Kimberley	4 (3%)
Midwest	4 (3%)
Pilbara	3 (2%)
South West	19 (12%)
Wheatbelt	7 (5%)
Primary roles ^a^ (*n* = 149)	
Pharmacy Owner	44 (29%)
Pharmacist Manager	30 (20%)
Pharmacist in Charge	35 (23%)
Pharmacist	54 (36%)
Locum Pharmacist	9 (6%)
Intern Pharmacist	11 (7%)
Other	3 (2%)
Years of practice (*n* = 149)	
Less than 1 year	4 (3%)
1–5 years	31 (23%)
6–10 years	22 (16%)
11–15 years	22 (16%)
16–20 years	18 (13%)
More than 20 years	39 (29%)
Hours of practice in community pharmacy (per week) (*n* = 149)	
Less than 10 h	6 (4%)
10–20 h	12 (8%)
20–30 h	21 (14%)
30–40 h	61 (41%)
More than 40 h	46 (31%)
Varies greatly	2 (1%)
No longer practicing	1 (1%)

^a^ Multiple selection options were allowable; therefore, the total percentage exceeds 100%.

## Data Availability

The data presented in this study are available on request from the corresponding author. The data are not publicly available due to ethical and privacy restrictions.
